# A 3D-Printed, High-Fidelity Pelvis Training Model: Cookbook Instructions and First Experience

**DOI:** 10.3390/jcm13216416

**Published:** 2024-10-26

**Authors:** Radu Claudiu Elisei, Florin Graur, Amir Szold, Răzvan Couți, Sever Cãlin Moldovan, Emil Moiş, Călin Popa, Doina Pisla, Calin Vaida, Paul Tucan, Nadim Al-Hajjar

**Affiliations:** 1 Department of Surgery, “Iuliu Hatieganu” University of Medicine and Pharmacy, 400012 Cluj-Napoca, Romania; radu_elisei@yahoo.com (R.C.E.); drmoisemil@gmail.com (E.M.); calinp2003@yahoo.com (C.P.); na_hajjar@yahoo.com (N.A.-H.); 2 Emergency Clinical County Hospital, 420016 Bistrita, Romania; razvan.couti@gmail.com (R.C.); severcalinmoldovan@gmail.com (S.C.M.); 3 “Octavian Fodor” Regional Institute of Gastroenterology and Hepatology, 400394 Cluj-Napoca, Romania; 4 CESTER Department, Faculty of Industrial Engineering, Robotics and Production Management, Technical University of Cluj-Napoca, 400114 Cluj-Napoca, Romania; doina.pisla@mep.utcluj.ro (D.P.); calin.vaida@mep.utcluj.ro (C.V.); paul.tucan@mep.utcluj.ro (P.T.); 5 Assia Medical, Assuta Medical Centre, Tel Aviv 6971028, Israel; amikisz@gmail.com; 6 Sheba Medical Centre and School of Medicine, Tel Aviv University, Tel Aviv 6997801, Israel

**Keywords:** anatomic pelvic trainer, versatile pelvi-trainer, box-trainer, 3D printing, training, colorectal surgery training, laparoscopic surgery training

## Abstract

**Background:** Since laparoscopic surgery became the gold standard for colorectal procedures, specific skills are required to achieve good outcomes. The best way to acquire basic and advanced skills and reach the learning curve plateau is by using dedicated simulators: box-trainers, video-trainers and virtual reality simulators. Laparoscopic skills training outside the operating room is cost-beneficial, faster and safer, and does not harm the patient. When compared to box-trainers, virtual reality simulators and cadaver models have no additional benefits. Several laparoscopic trainers available on the market as well as homemade box and video-trainers, most of them using plastic boxes and standard webcams, were described in the literature. The majority of them involve training on a flat surface without any anatomical environment. In addition to their demonstrated benefits, box-trainers which add anatomic details can improve the training quality and skills development of surgeons. **Methods:** We created a 3D-printed anatomic pelvi-trainer which offers a real-size narrow pelvic space environment for training. The model was created starting with a CT-scan performed on a female pelvis from the Anatomy Museum (Cluj-Napoca University of Medicine and Pharmacy, Romania), using Invesalius 3 software (Centro de Tecnologia da informação Renato Archer CTI, InVesalius open-source software, Campinas, Brazil) for segmentation, Fusion 360 with Netfabb software (Autodesk software company, Fusion 360 with Netfabb, San Francisco, CA, USA) for 3D modeling and a FDM technology 3D printer (Stratasys 3D printing company, Fortus 380mc 3D printer, Minneapolis, MN, USA). In addition, a metal mold for casting silicone valves was made for camera and endoscopic instruments ports. The trainer was tested and compared using a laparoscopic camera, a standard full HD webcam and “V-Box” (INTECH—Innovative Training Technologies, Milano, Italia), a dedicated hard paper box. The pelvi-trainer was tested by 33 surgeons with different qualifications and expertise. **Results:** We made a complete box-trainer with a versatile 3D-printed pelvi-trainer inside, designed for a wide range of basic and advanced laparoscopic skills training in the narrow pelvic space. We assessed the feedback of 33 surgeons regarding their experience using the anatomic 3D-printed pelvi-trainer for laparoscopic surgery training in the narrow pelvic space. Each surgeon tested the pelvi-trainer in three different setups: using a laparoscopic camera, using a webcam connected to a laptop and a “V-BOX” hard paper box. In the experiments that were performed, each participant completed a questionnaire regarding his/her experience using the pelvi-trainer. The results were positive, validating the device as a valid tool for training. **Conclusions:** We validated the anatomic pelvi-trainer designed by our team as a valuable alternative for basic and advanced laparoscopic surgery training outside the operating room for pelvic organs procedures, proving that it supports a much faster learning curve for colorectal procedures without harming the patients.

## 1. Introduction

Laparoscopic surgery has been the gold standard approach for colorectal cancer for over a decade [1], with rectal cancer representing almost a third of all CRCs (colorectal cancer) [2,3]. Laparoscopic rectal surgery demands advanced laparoscopic skills due to the difficult dissection and TME (total mesorectal excision) in the narrow pelvic space [1]. At the same time, robotic rectal surgery has great advantages over laparoscopic surgery, such as having a wide range of movements (seven degrees of freedom) due to wrist instruments [1]. And in between both of these types of surgery, there are robotized instruments, which are laparoscopic instruments with advantages of robotic ones [4,5].

For every procedure, each surgeon must go through the learning curve to reach a level of experience where there is a low complication rate. For each surgical procedure, there are a number of 15 to 100 procedures [6]. In laparoscopic surgery, it is faster, easier, safer and more cost-effective to reach the learning curve plateau by training outside the operating room in order to achieve the basic and advanced skills for specific procedures [7,8]. Teaching residents in the operating room is costly, unethical and time-consuming [9], whereas using different simulators creates the basic skill for complex interventions in a safe and controlled way.

Halsted’s training principle “see one, do one, teach one” cannot be applied to laparoscopic surgery because the trainee cannot see the surgical field, the instruments and the operator hands at the same time [9]. Laparoscopic surgery training began in Europe in the 1980s in Kiel (Semm courses) and Paris—“French Connection”—(Mouret and Dubois) and Bordeaux (Perissat) [7,10,11].

Laparoscopic surgery training outside the operating room can be achieved by using animate models (i.e., a porcine model) or cadaver models [7,12,13] and inanimate models and simulators: box-trainers, video-trainers and virtual reality simulators [7,14,15,16,17]. Furthermore, training outside the OR (operating room) for advanced laparoscopic skills will improve the learning curve; for example, to achieve a high degree in CRS, it is estimated that a surgeon needs to be involved around 60 procedures [18,19,20].

There are two types of simulation training: self-regulated and instructor-regulated. When self-regulated training was compared to no other types of training, the outcomes were favorable, and there were only moderately favorable outcomes when compared to non-simulation training, i.e., video surgical instructions [10]. According to Zendejas B et al., there is no significant difference between self-regulated and instructor-regulated training [10].

Studies showed equivalence in performance outcomes when comparing box-simulators (bench models) to cadaver models (costly and appropriate use concerns) and with live animal models (costly, require specialized personnel and appropriate use concerns), which make box-trainers also a great predictor of trainee technical skills [21,22,23]. Also, no significant difference was found between 2D and in 3D laparoscopic imaging regarding, for example, the intracorporeal suturing [21,24,25].

Minimally invasive surgery requires basic, special and advanced skills: hand–eye coordination, depth perception, ambidexterity, adaptation to a 2D image/environment, adaptation to a magnified image, instrument to target accuracy, handling long instruments and the fulcrum effect [21,26]. There are many exercises to be practiced in the box-trainers to acquire and master these skills: beads transfer, placing rings on rigid pegs or laces of appropriate color, rope transfer from one instrument to another from one end to the other, intracorporeal suturing and knot-tying (the needle positioning maneuver was found to be the most difficult, challenging and time-consuming in laparoscopic suturing training), precision cutting on a predetermined path and mesh placement, grape decortication and passing a stitch with needle through a predetermined path of rings [6,9,21,22,27].

Munz et al. [28] made a randomized study to compare the box-trainer to a VR simulator to determine their efficiency in basic laparoscopic skills and found no difference between them.

Box-trainers have several advantages over VR simulators: the preservation of haptic feedback, an affordable cost, the use of standard laparoscopic instruments and the possibility of training at home [29,30,31]. Classic box-trainers remain the best option in the matter of cost-effectiveness and have a very important role in basic and advanced laparoscopic skills training [29].

Since the first laparoscopic simulator was produced by Sackier et al. [32] in 1991, many authors have described several homemade and/or low-cost laparoscopic simulators for laparoscopic skills training [33,34,35,36,37,38,39]. But there is no anatomical and/or 3D-printed pelvi-trainer for training basic and advanced laparoscopic skills in the narrow pelvic space.

The aim of this study was to create a 3D-printed anatomic pelvi-trainer, starting from a CT-scan, as a central element of a box/video-trainer to be used for basic and advanced training in pelvic organs minimally invasive surgery and evaluate it by collecting the responses of surgeons using an online questionnaire.

## 2. Materials and Methods

The study was conducted at the Emergency Clinical County Hospital from Bistrita, Romania, with the approval of the Ethical Council No. 5356 of 27 May 2022.

### 2.1. Making the Anatomical Pelvi-Trainer

#### 2.1.1. First Prototype

##### Virtual Pelvi-Trainer

**Figure 1:**
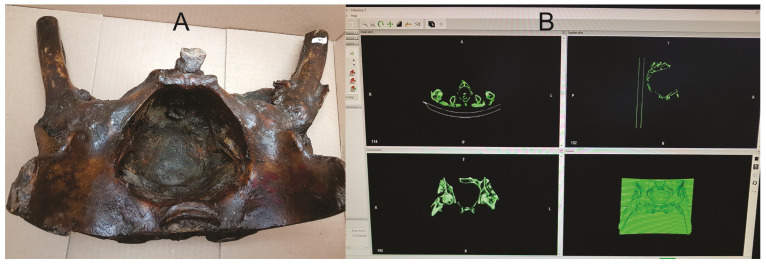
(**A**) Plastified pelvis from the Anatomy Museum; (**B**) segmentation and 3D reconstruction using Invesalius 3 software.

We performed a CT-scan (64 slices at 1.25 mm slice thickness using GE Revolution EVO X-Ray system GMDN code: 37618, GE Healthcare Japan Corporation, Tokyo, Japan) to a plastified female pelvis from the Anatomy Museum of “Iuliu Hatieganu” University of Medicine and Pharmacy from Cluj-Napoca, Romania (Figure 1A). Using the DICOM file, we performed an accurate segmentation of the object using an open-source segmentation software—InVesalius 3 (Centro de Tecnologia da informação Renato Archer CTI, InVesalius open-source software, Brazil) and obtained a stereolithography (STL) file (Figure 1B).

We opened the DICOM images in the segmentation software, and by using the “region growing” function, we selected the whole object; using the “crop” or “delete” buttons we removed the unwanted regions.

To create a smooth surface for the model, we used the “smooth” button from the “surface” menu.

To close the holes of the 3D model, we used the “close holes” button and then we generated the first STL file.

##### Physical Pelvi-Trainer

**Figure 2:**
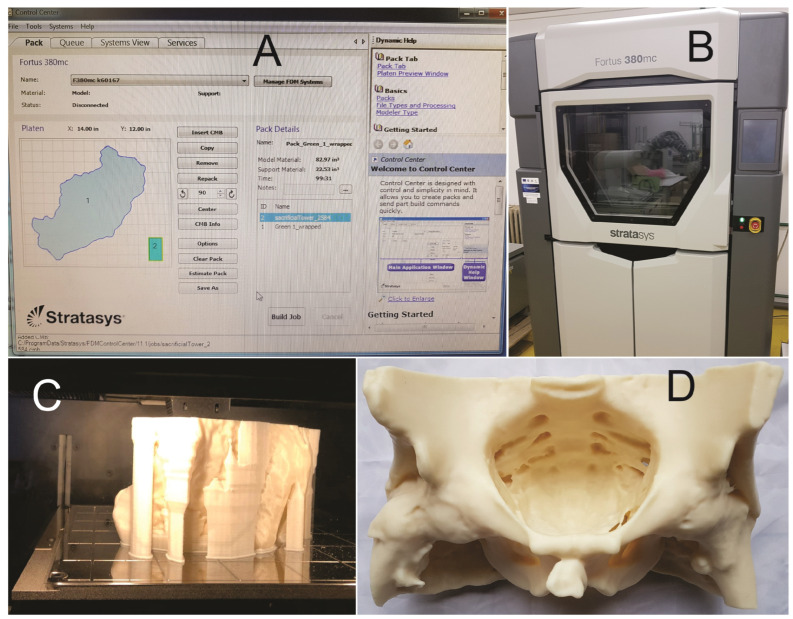
(**A**) 3D printer Control Center with object data; (**B**) StratasysFortus 380mc 3D printer; (**C**) 3D printing process; (**D**) pelvis after support material was removed.

We 3D-printed the first STL file (Figure 2A,C) using a professional 3D printer (Figure 2B) Stratasys Fortus 380mc (the FDM—fused deposition modeling technology—Stratasys 3D printing company, Fortus 380mc 3D printer, Minneapolis, MN, USA). The model was printed using acrylonitrile butadiene styrene material (ABS) with a layer thickness of 0.1778 mm within the CESTER research laboratory of the Technical University of Cluj-Napoca, Romania. The printing job was completed in 99 h and 31 min, using 1359.64 cm^3^ of model material and 369.2 cm^3^ of support material to create the 3D pelvic model.

We tried to use this first prototype for training purposes but it was not proper because of its instability on a flat surface and because of the thickness of the pelvis walls (Figure 2D), so we decided to go further with the 3D modeling of the first prototype STL file.

#### 2.1.2. Second Prototype

##### Virtual Pelvi-Trainer

**Figure 3:**
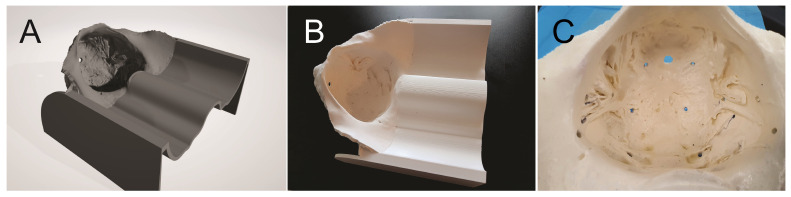
(**A**) Virtual pelvi-trainer; (**B**) 3D-printed pelvi-trainer; (**C**) pelvic space prepared for exercises.

We opened the first STL file in “Autodesk Fusion 360 with Netfabb”, a 3D modeling software (Autodesk software company, Fusion 360 with Netfabb, CA, USA) and we created the virtual anatomical pelvi-trainer (Figure 3A):

We selected the inner surface of the 3D model using the “region growing” button and using the “delete” button we removed the remaining regions.

By using the “extrude” function, we multiplied the last cranial line of the 3D model and extended it to recreate the lumbar spine and the posterior abdominal wall with 13 cm, and using the same function, starting from the lateral sides of the new created part, we created geometries downwards, creating the supports of the model.

Using the “automatic repair” function, we corrected the possible integrity problems of the model.

We optimized the number of triangles on the surface of the model using the “reduce” function for improving the file performance for 3D printing.

Using the “extrude” function again, we defined a 5 mm thickness to the outer surface of the 3D model.

Using the “cut” function, a 10 mm hole was created where the anus should be, to be able to fix the ex vivo porcine rectum inside the pelvis.

We generated the second STL file by using the “export” function.

##### Physical Pelvi-Trainer

Using the same 3D printer, model material, support material and the same layer thickness, we 3D-printed the second pelvi-trainer prototype. The printing job was completed in 72 h and 22 min, using 685.031 cm^3^ of model material and 461.66 cm^3^ of support material; after dissolving the support material, the result was a 3D-printed anatomic pelvi-trainer with a length of 277.2 mm, a width of 250 mm and a height of 152.4 mm, with a 1:1 scale of the inner pelvic space (Figure 3B). The ABS printed using the FDM technology is a porous material which allows liquids to infiltrate through it. To prevent liquid infiltration into the 3D-printed material, we sealed the entire surface of the model with industrial acetone based varnish for plastics. We made 4 holes in a square shape at 6 cm one to another to the base of pelvic space, 6 holes in a “U” shape on the lateral and front part of the pelvic space at 4 cm one to another and another 2 holes at 3 cm from the “U” shape end holes with an electric drilling machine using a 3.5 mm drilling bit to be able to prepare the pelvi-trainer for laparoscopic training exercises (Figure 3C).

### 2.2. Making the Silicon Valves

**Figure 4:**
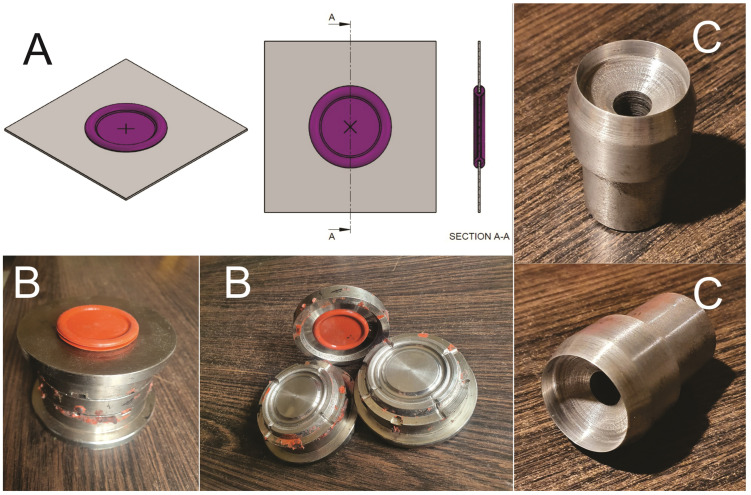
(**A**) Virtual design of the silicone valves; (**B**) metal mold with silicone valve; (**C**) metal cutting device for valves holes in the box lid.

To be able to mimic the abdominal wall and insert the trocars, there is need for silicone valves on the lid of the box-trainer. On the market, we could not find silicon valves alone to purchase for this purpose and could only find them with entire box/video-trainers, with prices from USD 200 [40,41]. Therefore, we came up with a project to produce these silicone valves (Figure 4A) and we developed a 3-segments metal mold to produce these silicon valves (Figure 4B) that can be assembled perfectly on the 38 mm round holes, made with a special metal cutting device (Figure 4C) (like one to make leather eyelets) on the plastic box lid. The diameter of the valves is 38 mm to offer a large range of freedom of degrees for instruments and cameras, with a minimum of 30° for the plastic lid, mimicking the behavior of the human abdomen.

The inner part of the silicone valves is 2 mm thick and 39 mm in diameter with the outer diameter of 47 mm. In the center of the valves, we made 4 mm or 8 mm holes with a leather eyelets device in order to be able to insert 5 mm or 10 mm trocars through them.

### 2.3. Assembling the Box-Trainer

**Figure 5:**
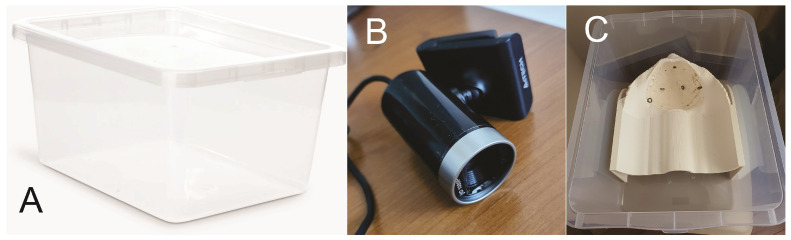
(**A**) Plastic storage box; (**B**) A4Tech PH-910H webcam; (**C**) pelvi-trainer inside the plastic box.

To assemble the box-trainer, we used a 20 liter transparent storage box of 43/33/21 cm (L/W/H) (Figure 5A) purchased for EUR 5 from a dedicated store [42]. Also, we purchased a standard full HD webcam (Figure 5B) (A4Tech PK-910H, A4TECH®, Taipei, Taiwan) from an online store for EUR 27 with a minimum of 30 fps (frames per second) for a minimum delay, which can be fixed with a screw to the lid inside the box [43]. The 3D-printed pelvi-trainer fits perfectly laterally into the storage box (Figure 5C). The remaining space from the pelvi-trainer to the other end of the box was left unused.

**Figure 6:**
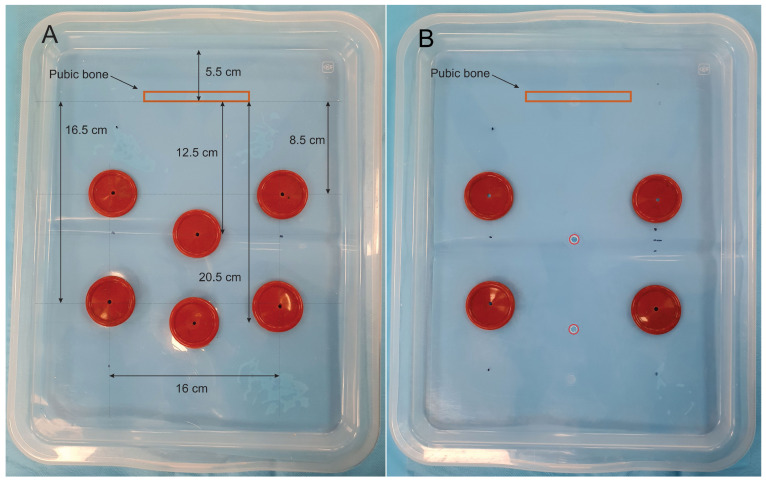
(**A**) Box lid for laparoscopic camera applications; (**B**) box lid for webcam applications.

We prepared 2 box lids for the two different visualization options of the pelvi-trainer, one with a laparoscope and the other with a webcam:

For laparoscope usage within the box-trainer, we made 6 holes of 38 mm using the metal cutting device, as follows: 2 in the middle line for the laparoscope and 4 (2 on each side) for the surgical instruments. We assembled a silicone valve in each hole. The laparoscopic camera valves center is at 18 cm and 26 cm from the box front edge, respectively, and at 12.5 cm and 20.5 cm from the pelvi-trainer pubic bone; the distance from the left and right instrument ports is 16 cm, with a distance of 14 cm and 22 cm from the box front edge on each side (Figure 6A).

For the webcam positioning on the box-trainer, we made 2 holes of 8 mm with an electric drilling machine using an 8 mm drilling bit for fixing the webcam with an 8 mm screw at 18 cm and 26 cm from the box front edge and 4 holes of 38 mm using the metal cutting device (2 on each side) for laparoscopic instruments and we attached silicone valves on those 4 holes. The instrument ports valves are positioned like the ones for the laparoscopic camera (Figure 6B).

### 2.4. Testing and Validating the Pelvi-Trainer

**Figure 7:**
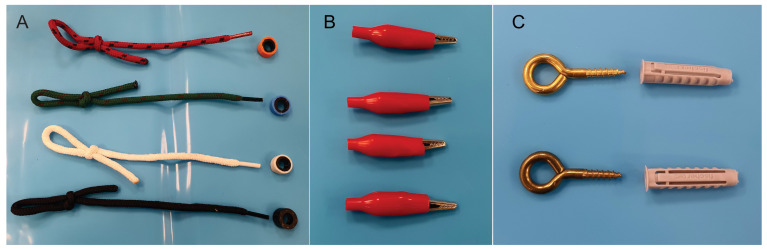
(**A**) Colour laces and rings for Exercise 1, (**B**) crocodile clamps for preparing Exercise 3 and 4; (**C**) ring screws and plastic dowels for preparing Exercise 2.

To test and evaluate the box-trainer, we defined four basic exercises: “rings on laces”, “string threading”, “pattern cutting”, “suture drill” and we prepared the pelvi-trainer for each exercise (Figure 7):

Exercise 1: “rings on laces”

**Figure 8:**
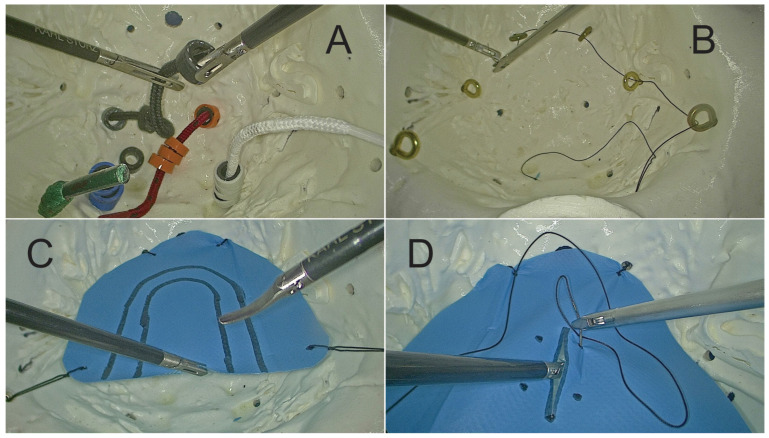
(**A**) Exercise 1; (**B**) Exercise 2; (**C**) Exercise 3; (**D**) Exercise 4.

- Task: Place 16 colored rings (4 of each color) on 4 laces of similar colors using an endoscopic needle holder in the dominant hand and an endoscopic grasper or a Maryland dissector in the other hand (Figure 8A).
- Preparing the pelvi-trainer: Through the holes on the bottom of the pelvis, we inserted 4 round color laces (3.5 mm thick) with a length of 10 cm inside the pelvis and a stopping knot on the outside and we prepared colored rings by cutting 3 mm thick slices of 4 different color pressure hose using two endoscopic graspers or one endoscopic grasper and one Maryland dissector.

Exercise 2: “string threading”

- Task: Put a 3-0 silk suture with a curved needle through all the 8 rings from right to left and then from left to right using an endoscopic needle holder in the dominant hand and an endoscopic grasper or a Maryland dissector in the other hand (Figure 8B).
- Preparing the pelvi-trainer: We installed 8 ring screws of 3 mm diameter and 16 mm length [44] through the “U” shape holes made on the lateral and front sides of the pelvic space and we fixed them on the outside with 5 mm diameter and 2 mm length plastic dowels [45].

Exercise 3: “pattern cutting”

- Task: Cut the drape between the two “U” shape lines using endoscopic scissors in the dominant hand and an endoscopic grasper or a Maryland dissector in the other hand (Figure 8C).
- Preparing the pelvi-trainer: Inside the pelvic space, we installed a “U” shape drape with 2 “U” shape lines on it and we fixed it with silk stitches on the outside using “crocodile shape” clamps [46] (Figure 8D).

Exercise 4: “suture drill”

- Task: Repair the incision with two sutures with intracorporeal knots and secure each knot twice using a 3-0 silk suture on a curved tapered needle using a laparoscopic needle holder on the dominant hand and an endoscopic grasper or a Maryland dissector on the other hand.
- Preparing the pelvi-trainer: Inside the pelvic space, we installed a “U” shape drape with a 3 cm incision in the middle and 2 suture points on each side of the incision and we fixed it with 4 silk stitches on the outside using “crocodile shape” clamps [46].

**Figure 9:**
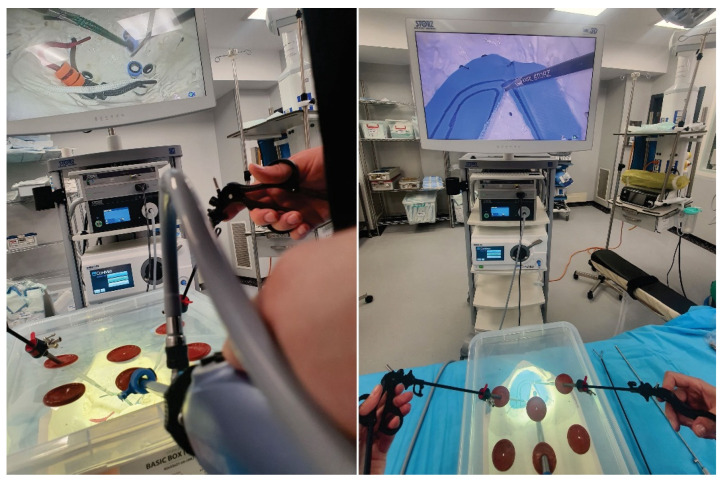
Applications for the 3D-printed pelvi-trainer: using laparoscopic camera.

**Figure 10:**
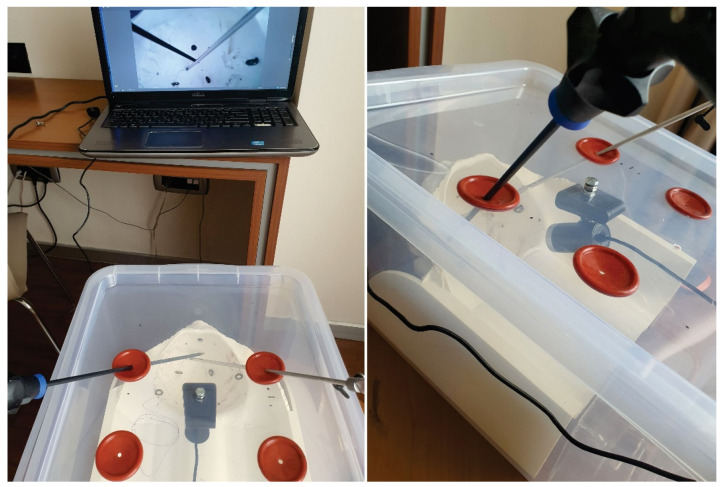
Applications for the 3D-printed pelvi-trainer: using a webcam and a laptop/tablet.

**Figure 11:**
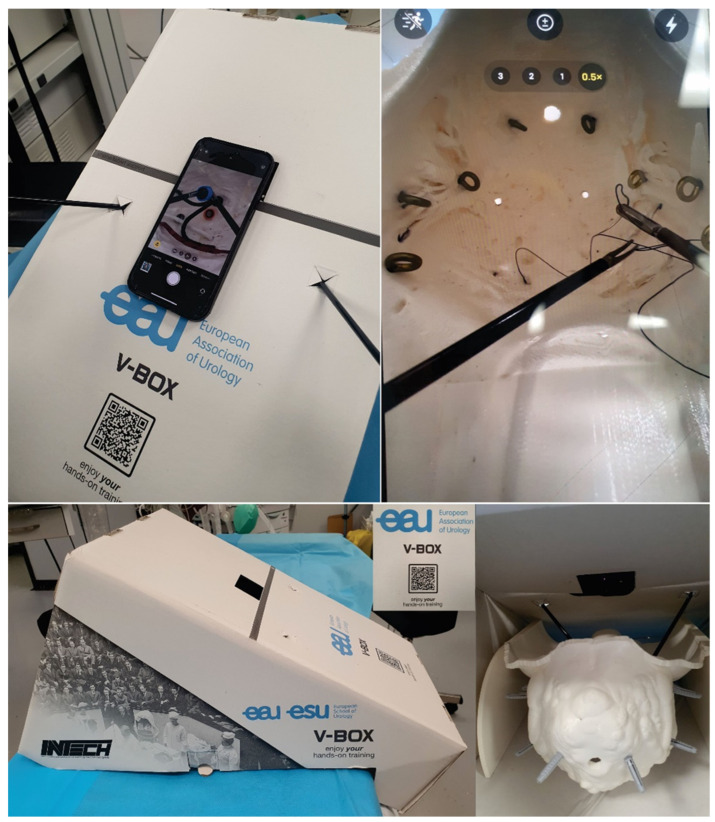
Applications for the 3D-printed pelvi-trainer: using the “V-BOX”, a dedicated hard paper box designed by INTECH (Innovative Training Technologies) for training purposes.

All exercises were performed by 33 surgeons (11 consultant general surgeons, 9 specialist general surgeons, 7 resident surgeons, 2 consultant urology surgeons, 3 specialist urology surgeons and 1 resident gynecology surgeon) using the pelvi-trainer with a laparoscopic camera (Figure 9), a webcam (Figure 10), using the “V-box” (Figure 11), a hard paper box designed for hands-on training by INTECH (Innovative Training Technologies) [47] in collaboration with the European Association of Urology and a smartphone (or tablet) (Table 1). After performing the exercises, all 33 participants completed an assessment questionnaire with 12 Likert scale questions (Q1–12), 1 multiple choice question (Q13), 3 single choice questions (Q14–16) and 2 questions that require suggestions to be written for improving the pelvi-trainer and for exercises to be performed inside the pelvi-trainer (Q17–18) (Table 1). They evaluated the anatomic 3D-printed pelvi-trainer designed by our team from a qualitative point of view and compared it to the flat surface box-trainers usually used for basic laparoscopic training.

## 3. Results

We made a versatile anatomic 3D-printed pelvi-trainer at a 1:1 scale of a female pelvis as the main part of a box-trainer for training in laparoscopic surgery for basic and advanced skills, with a 3D printing time of 72 h 22 min. This pelvi-trainer can be used with a laparoscopic camera, with a webcam, or in a dedicated hard paper box (INTECH “V-box” [47]) using the camera of a smartphone or a tablet.

The 3D-printed pelvi-trainer prototype overall cost (materials and production) is EUR 875 (NUtechnologies Ltd., Timișoara, Romania) and the overall cost of the box-trainer with all of the accessories for the four exercises is around EUR 1000.

After the assessment of the 33 answers to the questionnaire, we found out that the 3D-printed pelvi-trainer designed by our team is very useful and feasible to be used for basic and advanced laparoscopic skills training. The doctors who tested the pelvi-trainer answered to 18 questions regarding their experience using the anatomic 3D-printed box-trainer.

**Figure 12:**
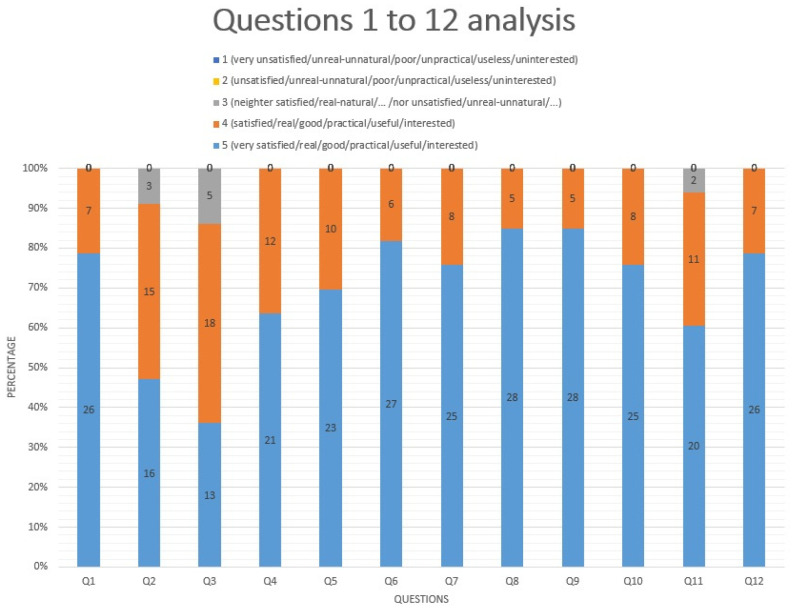
Questionnaire assessment: questions 1 to 12 analysis. (The numbers in the chart are absolute numbers).

Of the participants who tested the pelvi-trainer, 100% answered nine questions, 94% answered Q11, 91% answered Q2 and 85% answered Q3 with grade 5 or 4 regarding their experience using the anatomical pelvi-trainer (Figure 12).

For Q14 and Q16, all 33 participants answered that an anatomical 3D-printed pelvi-trainer is preferred to the flat surface ones and they would participate in a hands-on course with exercises using this pelvi-trainer.

**Figure 13:**
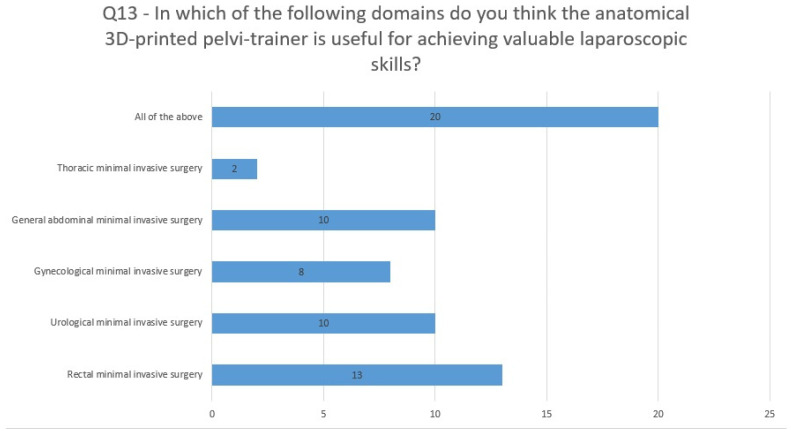
Questionnaire assessment: question 13 analysis. (The numbers in the chart are absolute numbers).

For Q13 (In which of the following areas do you think the anatomical 3D-printed pelvi-trainer is useful for achieving valuable laparoscopic skills?), 39% considered the anatomical pelvi-trainer useful for training in rectal minimally invasive surgery and 30% found it useful for training in urology and gynecology minimally invasive surgery, while more than 60% answered that it can be used for training in all options listed in the question (Figure 13).

**Figure 14:**
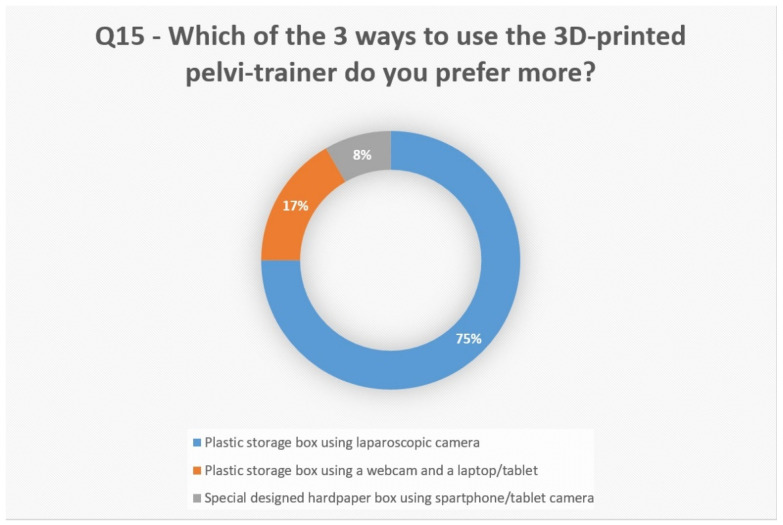
Questionnaire assessment: question 15 analysis.

When asked (Q15) in which way they prefer to use the pelvi-trainer, 75% answered “plastic storage box using laparoscopic camera” (Figure 14).

When participants were asked (Q17) for suggestions to improve the anatomical 3D-printed pelvi-trainer, they gave us a lot of valuable answers: a self-sustaining laparoscopic camera holder, the use of “fresh meat”, more diversified training models, ureters and vessels inside the pelvis, the real color of pelvic environment and 3D-printed/artificial organs to operate on inside the pelvi-trainer. When they were asked (Q16) what other applications/exercises they would like to test using the pelvi-trainer, we obtained many valuable answers: all kinds of pelvic surgeries, anastomosis between ureter and bladder, similar exercises that reproduce the robotic technique, gynecological pathologies, hysterectomy, anexectomy, lymph node sampling, simulating inguinal hernia repair, prostatectomy model, vascular dissection and anastomosis, robotic surgery training, rectal resection using staplers and difficult procedures.

## 4. Discussion

The 3D-printed pelvi-trainer designed by our team is the only anatomic pelvi-trainer developed and published until now which allows acquiring laparoscopic skills in the narrow pelvic space. The cost of the 3D-printed pelvi-trainer prototype represents over 90% of the overall cost of the entire box-trainer. With mass production and not using 3D printing, the overall cost can be decreased by at least 60% and it can be adjusted for many exercises including operation on ex vivo pig pelvic organs (i.e., rectum, bladder, prostate). Also, it enables the use of 3D-printed artificial organs (i.e., 3D-printed vessels, ureters, bladder, prostate, rectum, covered by artificial fat tissue and peritoneum) and simulates real surgeries for advanced laparoscopic surgical training. It also enables training for robotic surgery as a complement to the actual training programs [48]. In the future, our pelvi-trainer can be tested for training using experimental robotic systems [49,50].

The distance between the umbilical scar and pubic bone is 15 cm for people with 145 to 178 cm stature [51]. In our pelvi-trainer, we do not have the tissue between the peritoneum and the pubic bone, and we decided that a distance of 12.5 cm from the optic trocar and pelvi-trainer pubis is acceptable, and with another port available at 20.5 cm from the pubic bone it offers good visibility of the entire pelvi-trainer; the distance to the target organ/zone is between 10 and 20 cm, as shown by Supe AN et al. [52]. At the same time, we have to know, when we place the umbilical port, that the placement of the umbilicus in overweight and obese patients is situated with 1.2 to 3.5 cm below the abdominal midline [53]. Also, the umbilicus is situated usually cephalad or at aortic bifurcation and at the point where the left common iliac vein crosses the midline [54].

According to Maathews CA et al. [55], in women with an umbilicus–pubis distance less then 16 cm, they require port placement above the umbilicus for robotic gynecologic surgery. Also, as a woman ages, the umbilicus usually has a lower position because they lose abdominal muscle tone [55].

The operating ports of our box-trainer are positioned at 8 cm from the optic trocar. Supe AN et al. [52] have shown that the optimal distance of the operating ports is 7 cm from the central port. The working angle between the instruments in our box-trainer is 65°–70°. The optimal working angle between the instruments in laparoscopy is 60°–90° according to Supe AN et al. [52] and the manipulation angle should range between 45° and 75° according to Yeola (Pate) ME et al. [56].

According to Sakamoto A et al. [57], the distance between the elevated periumbilical skin and lumbar spine is 15 cm and 13 cm from the peritoneum to the lumbar spine in non-obese patients. The distance between the box-trainer lid and 3D-printed pelvi-trainer lumbar spine is 8.5 cm. Because in our case we do not have any other tissue above the lumbar spine, we had to consider the distance acceptable for training. In the case of training using ex vivo pig pelvic organs, it is recommended to use a higher box.

When operating on real patients, it is mandatory to know the regional anatomy but also it is very important to know the possible anatomical variations of important structures (arteries, veins, nerves, ureter). A very well documented and illustrated article for the anatomical variations of pelvic structures was published by Kostov S et al. [58]. Also, the vascular anatomy of the abdominal wall and its variations are very important for avoiding injuries of important vessels or nerves when placing the trocars, as well-described by Kostov S et al. [59].

Most minimally invasive surgery complications occur during entry into the abdominal cavity. A quarter of the complications are caused by energy-based surgical devices, including bowel and ureter injuries. Most of them are completely preventable. Also, their delayed diagnosis increases mortality and morbidity [60]. This is another reason for a good training of residents and young specialists, also using energy-based surgical devices.

The silicone valves designed by our team for the trocar ports of the box-trainer are very versatile. They can be used on any box-trainer and made of any material with a lid thickness of 1.5–2 mm with a production cost less than EUR 4 each. With a 38 mm diameter, they offer a very good maneuverability and freedom of movement for trocars and endoscopic instruments.

Several articles were published about homemade box-trainers [29,33,34,35,36,37,38,39]. Also, you can find several training simulators models to purchase [40,47,61]. All of them propose exercises which slightly differ from one another but all use a flat surface at the base of the box. None of them propose an anatomic actual size pelvic space environment for training. Most of them use plastic storage boxes and use standard webcams [34,35,36,38,39] and one uses a homemade wooden box; for lighting and vision, this latter article uses an LED strip and a bullet mini camera [33]. However, none are described as feasible to be used with a laparoscopic camera or using a smartphone or tablet camera. The only 3D-printed, anatomic pelvi-trainer designed so far which offers the possibility of training outside the operating room (OR) in a real-size narrow pelvic environment is the pelvi-trainer designed by our team, which can be used with a laparoscopic camera, standard webcam or smartphone/tablet camera. For a better quality image and minimally delay, we recommend using a full HD webcam with at least 60 fps. In addition, our pelvi-trainer is suitable to be used for operations of ex vivo pelvic organs outside the OR for advanced laparoscopic surgery training.

According to Bridges M et al. (a study made by the University of Tennessee Medical Center—Knoxville), for every graduate resident who was trained in the operating room (for 4 years of training) there was over 11.000 min of operating time lost with a cost of almost USD 48.000 [8].

Our box-trainer offers the possibility of acquiring the necessary basic and advanced skills for pelvic endoscopic surgery, even at home, at an affordable price, without endangering the patients. According to Lin et al. [20], residents who attended advanced laparoscopy training outside the OR favored participation in the OR without endangering the patients.

There are two types of learning using a box-trainer or a laparoscopic simulator: instructor-regulated and self-regulated, with no significant difference between them [10]. In a systematic review, Zendejas B. et al. showed that there is a significant difference with moderately/greater outcomes in favor of box-trainers simulators in comparison to virtual reality simulators [10].

When comparing box-trainers to VR-simulators, the first group had greater outcomes than the second group, especially when talking about trainee satisfaction and tasks time, and there was no real benefit found by adding haptic feedback to VR-simulators [10]. Our box-trainer offers a more realistic environment than a common flat working space box-trainer, is suitable for pelvic endoscopic surgery and is more affordable than a VR-simulator (i.e., an LAP-X laparoscopic simulator for costs USD 4.817 [62]).

Our box/video-trainer with an anatomic 3D-printed pelvi-trainer can be used for training in basic and advanced surgical skills for the minimally invasive surgery of pelvic organs, but not in complicated cases like adhesions from previous surgeries, pelvic endometriosis, etc. [60]. We cannot mimic these types of complicated cases yet.

Also, the 3D-printed pelvi-trainer developed by our team has some limitations in anatomical details because it was 3D-reconstructed from a CT-scan performed on a plastinated specimen, a process which caused a loss of fine structure details, like vascular structures or nerves. This limitation can be corrected in the future by using high-resolution scanning devices like magnetic resonance microscopy as an imaging source for the 3D reconstruction and 3D modeling of an anatomic trainer [63].

Endoscopic ultrasound is one of the main procedures used for rectal cancer staging [64]. Our pelvi-trainer can be easily customized for rectal endoscopic ultrasound training in the future using 3D-printed organs or ex vivo porcine organs.

The 3D-printed pelvi-trainer designed by our team can be a reliable tool for training in pelvic endoscopic surgery in medical training centers. Once a box-trainer is verified and validated as reliable for training, practicing endoscopic surgery and also objective assessment, it can become a tool with an important role at an institutional level for certification and revalidation [21]

The revalidation has four goals: patient care improvement, setting standards for laparoscopy/surgery practice, continued education in surgical/medical fields and ensuring that doctors should remain competent through their entire careers and the patients should be reassured of that [21,25].

For the complete validation of our versatile anatomic 3D-printed pelvi-trainer, there is a need for a comparative study between our box-trainer and a flat surface box-trainer with resident surgeons, young specialist surgeons and experienced laparoscopic surgeons.

## 5. Conclusions

This concept of a versatile anatomic 3D-printed pelvi-trainer can bring a significant contribution to the development of laparoscopic surgery training outside the OR and to the standardization of training in minimally invasive pelvic organs surgery. It is a reliable, functional and versatile training tool, which is valuable for both residents and specialist surgeons for acquiring the necessary skills in the narrow pelvic space, bringing also a major financial and practical benefit.

## Figures and Tables

**Table 1:** The assessment questionnaire.

Q1	How satisfied are you with the experience using the 3D-printed pelvi-trainer?
Q2	How real/natural did you think the 3D-printed pelvi-trainer was?
Q3	What do you think about the general appearance of the 3D-printed pelvi-trainer?
Q4	What do you think about the quality of the anatomical 3D-printed pelvi-trainer?
Q5	What do you think about the quality of silicone valves used for inserting the trocars?
Q6	How satisfied are you with performing exercises using the 3D-printed pelvi-trainer?
Q7	How satisfied are you with the entire box-trainer complex (storage box + silicone valves + 3d-printed pelvi-trainer)?
Q8	How well can you exercise laparoscopic skills using the 3D-printed pelvi-trainer?
Q9	How useful do you think the 3D-printed pelvi-trainer is for laparoscopic skills development?
Q10	How practical/suitable do you think this anatomical 3D-printed pelvi-trainer is to be used for operating on ex vivo porcine rectum/small bowel?
Q11	How practical/suitable do you think this anatomical 3D-printed pelvi-trainer is to be used for acquiring valuable skills for robotic surgery ?
Q12	If you have the opportunity in the future, how interested are you in participating in a course to operate on porcine rectum and small bowel in this anatomical 3D-printed pelvi-trainer?
Q13	In which of the following fields do you think the anatomical 3D-printed pelvi-trainer is useful for achieving valuable laparoscopic skills?
Q14	Which box-trainers did you find more useful: the one with anatomical real-size 3D-printed pelvi-trainer inside or flat surface ones?
Q15	Which of the 3 ways to use the 3D-printed pelvi-trainer do you prefer more?
Q16	Would you like to participate in a course for laparoscopic skills with multiple exercises using this 3D-printed pelvi-trainer?
Q17	What suggestions for improving the anatomical 3D-printed pelvi-trainer do you have?
Q18	What other applications/exercises would you like to test using the anatomical 3D-printed pelvi-trainer in a future course?

## Data Availability

The data presented in this study are available on request from the corresponding author (graurf@yahoo.com).
